# Differences in gut microbiota correlate with symptoms and regional brain volumes in patients with late-life depression

**DOI:** 10.3389/fnagi.2022.885393

**Published:** 2022-07-27

**Authors:** Chia-Fen Tsai, Chia-Hsien Chuang, Yen-Po Wang, Ya-Bo Lin, Pei-Chi Tu, Pei-Yi Liu, Po-Shan Wu, Chung-Yen Lin, Ching-Liang Lu

**Affiliations:** ^1^Faculty of Medicine, National Yang Ming Chiao Tung University, Taipei, Taiwan; ^2^Department of Psychiatry, Taipei Veterans General Hospital, Taipei, Taiwan; ^3^Institute of Information Science, Academia Sinica, Taipei, Taiwan; ^4^Institute of Brain Science, National Yang Ming Chiao Tung University, Taipei, Taiwan; ^5^Endoscopy Center for Diagnosis and Treatment, Taipei Veterans General Hospital, Taipei, Taiwan; ^6^Division of Gastroenterology, Taipei Veterans General Hospital, Taipei, Taiwan; ^7^Department of Medicine, Taipei Veterans General Hospital, Taipei, Taiwan; ^8^Institute of Philosophy of Mind and Cognition, National Yang Ming Chiao Tung University, Taipei, Taiwan; ^9^Department of Medical Research, Taipei Veterans General Hospital, Taipei, Taiwan; ^10^Department of Dietetics and Nutrition, Taipei Veterans General Hospital, Taipei, Taiwan

**Keywords:** brain-gut axis, brain image, elderly, mood disorder, *Enterobacter* and *Burkholderia*

## Abstract

Depression is associated with gut dysbiosis that disrupts a gut-brain bidirectional axis. Gray matter volume changes in cortical and subcortical structures, including prefrontal regions and the hippocampus, have also been noted in depressive disorders. However, the link between gut microbiota and brain structures in depressed patients remains elusive. Neuropsychiatric measures, stool samples, and structural brain images were collected from 36 patients with late-life depression (LLD) and 17 healthy controls. 16S ribosomal RNA (rRNA) gene sequencing was used to profile stool microbial communities for quantitation of microbial composition, abundance, and diversity. T1-weighted brain images were assessed with voxel-based morphometry to detect alterations in gray matter volume between groups. Correlation analysis was performed to identify the possible association between depressive symptoms, brain structures and gut microbiota. We found a significant difference in the gut microbial composition between patients with late-life depression (LLD) and healthy controls. The genera *Enterobacter* and *Burkholderia* were positively correlated with depressive symptoms and negatively correlated with brain structural signatures in regions associated with memory, somatosensory integration, and emotional processing/cognition/regulation. Our study purports the microbiota-gut-brain axis as a potential mechanism mediating the symptomatology of LLD patients, which may facilitate the development of therapeutic strategies targeting gut microbes in the treatment of elderly depressed patients.

## Introduction

Major depressive disorder (MDD) is a common mental disorder with core symptoms of depressive mood, diminished interests, and anhedonia, resulting in significant emotional, functional, and economic strain on both the individual and society (Otte et al., 2016). As the global society is aging, depression has become the most serious mental problem and a major public health concern among older adults (Rodda et al., 2011). It is also a major contributor to the overall global burden of disease and is reported to be the world's leading cause of disability (Disease et al., 2018). Treatment for late-life depression (LLD) shows a less favorable response to antidepressants than depression in younger adults, and many studies have explored various possible hypotheses to provide targets for developing novel treatments (Alexopoulos, 2019).

Traditionally, the mechanisms underlying the pathophysiology of depression are ascribed to disturbances in neurotransmitters, stress hormones, inflammatory cytokines, and neurotrophic factors (Hasler, 2010). Recently, the gut microbiota has been proposed as an innovative field for the development and management of depression (Liang et al., 2018). In addition to depression, many studies have shown that gut dysbiosis is involved in the pathogenesis of various neuropsychiatric disorders, such as schizophrenia, autism spectrum disorder, multiple sclerosis, Alzheimer's disease, and Parkinson's disease (PD) (Morais et al., 2021). Dysregulation in the bidirectional interactions between the gut microbiome and brain is reported to contribute to the pathophysiology of these neuropsychiatric disorders through neurological, metabolic, hormonal and immunological signaling pathways (Martin et al., 2018). In a rodent model, fecal microbiota transplantation with “depression microbiota” derived from patients with MDD produced depression-like behaviors in germ-free mice (Zheng et al., 2016). Differences in gut microbiota diversity, richness and composition at various taxonomic levels have been identified in adult MDD patients compared to healthy volunteers (Jiang et al., 2015; Kelly et al., 2016; Zheng et al., 2016; Lin et al., 2017). Alterations in the gut microbiota contribute to dysregulation along the microbiota-gut-brain axis from the ecological effects of the gut microbiome and central humoral effects from neuroendocrinological and neuroimmunological pathways (Liu et al., 2019). Despite these facts, there is a paucity of information on how changes in gut microbiota can have any effects on the brain, particularly in depressed patient populations (Liu et al., 2019).

Brain magnetic resonance imaging (MRI) is a non-invasive modality with the capability of investigating alterations in and pathologies related to brain structure, circulation, function, and neural metabolism *in vivo*, which could enhance the understanding of microbiota-gut-brain interactions (Kano et al., 2018). In a recent meta-analysis, MDD patients, relative to healthy volunteers, showed increased cortical thickness in the posterior cingulate cortex, ventromedial prefrontal cortex, and anterior cingulate cortex and decreased cortical thickness in the gyrus rectus, orbital segment of the superior frontal gyrus, and middle temporal gyrus (Li et al., 2020). One recent study has explored the link between gut microbiota and brain structure in LLD patients, showing positive associations between gray matter (GM) volume in hippocampus/amygdala and the abundance of *Ruminococcaceae, Oscillibacter*, and *Lachnospiraceae* at genus level (Lee et al., 2022). However, that study was limited by relatively small patient numbers (*n* = 16) and absence of control group. In the present study, we examined the hypothesis that alterations in gut microbiota are associated with differences in regional brain structures related to mood regulation among a larger cohort of LLD patients (*n* = 36) in comparison with controls. Second, we explored distinction in microbial composition and their correlations with clinical symptoms in LLD patients.

## Methods

### Participants

This study was conducted at the psychiatric outpatient clinic in a tertiary medical center. We recruited 36 patients who were over 50 years old and fulfilled the diagnosis of MDD according to the Diagnostic and Statistical Manual of Mental Disorders, 4th Edition (DSM-IV-TR) (American Psychiatric Association., and American Psychiatric Association, Task Force on DSM-IV, 2000), made by board-certified psychiatrists. Another 17 healthy controls were enrolled through poster advertisements. The exclusion criteria for both groups were having (i) a diagnosis of a major neurocognitive disorder; (ii) other major psychiatric comorbidity (such as schizophrenia or bipolar disorder); (iii) major physical comorbidities (such as history of organic gastrointestinal diseases, including liver cirrhosis, fatty liver disease, peptic ulcer, inflammatory bowel disease, or any malignancy); (iv) any known active bacterial, fungal, or viral infection; (v) a history of receiving antibiotics, prebiotics or probiotics within 90 days prior to enrollment; and (vi) a history of regular usage of laxatives, gastrointestinal tract surgery, appendectomy, or cholecystectomy in the preceding 1 year. The Mini-International Neuropsychiatric Interview was performed by psychiatrists to exclude any individuals with psychiatric illness in the control group (Sheehan et al., 1998). The study was conducted in accordance with the Declaration of Helsinki and was approved by the local ethics review committee. All participants provided written informed consent prior to participating in the study.

### Questionnaires

For the baseline assessment, we gathered sociodemographic data (e.g., sex, age, and education), anthropometric data (e.g., weight and height) and history of constipation (defined as fewer than 3 bowel movements/week) and diabetes mellitus. The weight and height of the participants were measured by an assisting nurse, and body mass index (BMI), defined as weight (in kilograms) divided by squared height (in meters), was calculated. All participants were assessed for depressive symptoms, general cognitive function, quality of sleep and diet pattern by the following questionnaires. The 17-item Hamilton Depression Rating Scale (HAMD) is a widely used, semistructured measure of the severity of depressive symptoms that is rated by clinicians (Hamilton, 1960). The Montreal Cognitive Assessment (MoCA) is a brief test to examine general cognitive function with scores ranging from 0 to 30 (Tsai et al., 2012). Sleep disturbance was evaluated using the Pittsburgh Sleep Quality Index (PSQI), which demonstrates good reliability and validity in evaluating sleep problems, and higher scores indicate poorer sleep quality (Buysse et al., 1989). Various types of food and beverages that were consumed during the previous 1 month and fell into one of nine categories were assessed by using a validated semiquantitative simplified food frequency questionnaire (SFFQ) (Huang et al., 2011).

### Brain image data acquisition and preprocessing

All brain images were acquired on a 3.0-T GE Discovery MR750 whole-body high-speed MRI device (Discovery MR750, GE Inc., USA). Automated shimming procedures were performed, and scout images were obtained. A high-resolution structural image was acquired in the axial plane using the fast spoiled gradient-echo (FSPGR) sequence (BRAVO) on GE equipment with parameters (repetition time [TR] = 12.23 ms, echo time [TE] = 5.18 ms, inversion time [TI] = 450 ms, and flip angle = 12°) and an isotropic 1-mm voxel (field-of-view (FOV) = 256 × 256). All images were acquired parallel to the anterior commissure–posterior commissure line. These slices covered the cerebellum of each participant. To minimize the generation of motion artifacts during image acquisition, each participant's head was immobilized with cushions inside the coil.

Individual high-resolution T1-weighted volumetric images were processed using Statistical Parametric Mapping (SPM12, Wellcome Institute of Neurology, University College London, UK) executed in Linux-based MATLAB 2020a (MathWorks, Natick, MA, USA) with default settings. In the current study, the detailed voxel-based morphometry (VBM) approach included the following: Data were first carefully checked by an experienced radiologist to rule out any scanner artifacts, motion problems, or gross anatomical abnormalities for each participant. After data checking and origin identification, the Segment Toolbox from SPM12 was applied to every T1-weighted image to extract tissue maps corresponding to GM and white matter (WM) and cerebrospinal fluid (CSF) in native space. To achieve a higher accuracy of registration across subjects, all native space tissue segments were imported into a rigidly aligned space and iteratively registered to group-specific templates that were generated from all structural images in this study through non-linear warping using the Diffeomorphic Anatomical Registration Through Exponentiated Lie Algebra (DARTEL) toolbox. These images were resampled to 1.5-mm isotropic voxels. Subsequently, the resliced images of GM and GM were registered to a subject-specific template using the DARTEL template-creation toolbox to improve intersubject alignment, and the normalization function of the toolbox was used to normalize the individual GM and WM images to Montreal Neurological Institute (MNI) space (1.5-mm isotropic voxel). Finally, the GM map of each subject was warped using their corresponding, smooth, and reversible deformation parameters to the custom template space and then to the MNI standard space. For the GM volume, the warped images of GM were modulated by calculating the Jacobian determinants derived from the special normalization step and by multiplying each voxel by the relative change in volume. The modulation step was performed to correct volume changes that might have occurred during non-linear normalization. The warped modulated GM images were smoothed through the convolution of an 8-mm full-width at half-maximum isotropic Gaussian kernel before tissue volume calculation and voxelwise group comparisons. The total intracranial volume (TIV) was determined as the sum of GM, WM, and CSF volumes.

### Microbiome data sequencing and preprocessing

Fresh stool specimens were self-collected from both groups and then stored in an RNA stabilizing reagent (RNALater) at −80 °C until further analysis. Bacterial DNA from the stool specimens was extracted, amplified, and sequenced, as previously described (Li et al., 2017). The 16S ribosomal RNA (rRNA) gene sequence libraries were generated using the V3-V4 (341F(CCTACGGGNGGCWGCAG)/805R(GACTACHVGGGTATCTAATCC)) primer region (Sinclair et al., 2015) and sequenced on an Illumina MiSeq sequencer (Illumina Inc., San Diego, CA, USA). We mixed cases and controls within each platform to reduce sequencing bias between cases and controls caused by batch effects.

### Data analysis

#### Sample sized calculation

The number of participants required in each group was predetermined before the study and calculated to be at least 13 on the basis of difference of major bacterial organism between MDD and control groups based on previous literature (Lin et al., 2017). This calculation was performed using the Power and Sample Size Program (version 3.0.43) (Dupont and Plummer, 1990). The estimated parameters used to reject the null hypothesis included the population means of the experimental and control groups being equal with a probability (power) of 0.8, and the type I error probability associated with this test's null hypothesis was 0.05.

#### Demographic and behavioral data analysis

Categorical variables were compared using chi-square tests, and continuous variables were compared using Student's *t*-tests. All tests were based on two-tailed alternatives. For all variables, significance was defined as a two-tailed *p* value of < 0.05. All data processing and statistical analyses were performed using Statistical Package for Social Science software version 17 and Statistical Analysis Software (version 9.1, SAS Institute, Cary, NC, USA).

#### Brain image data analysis

**Figure 1:**
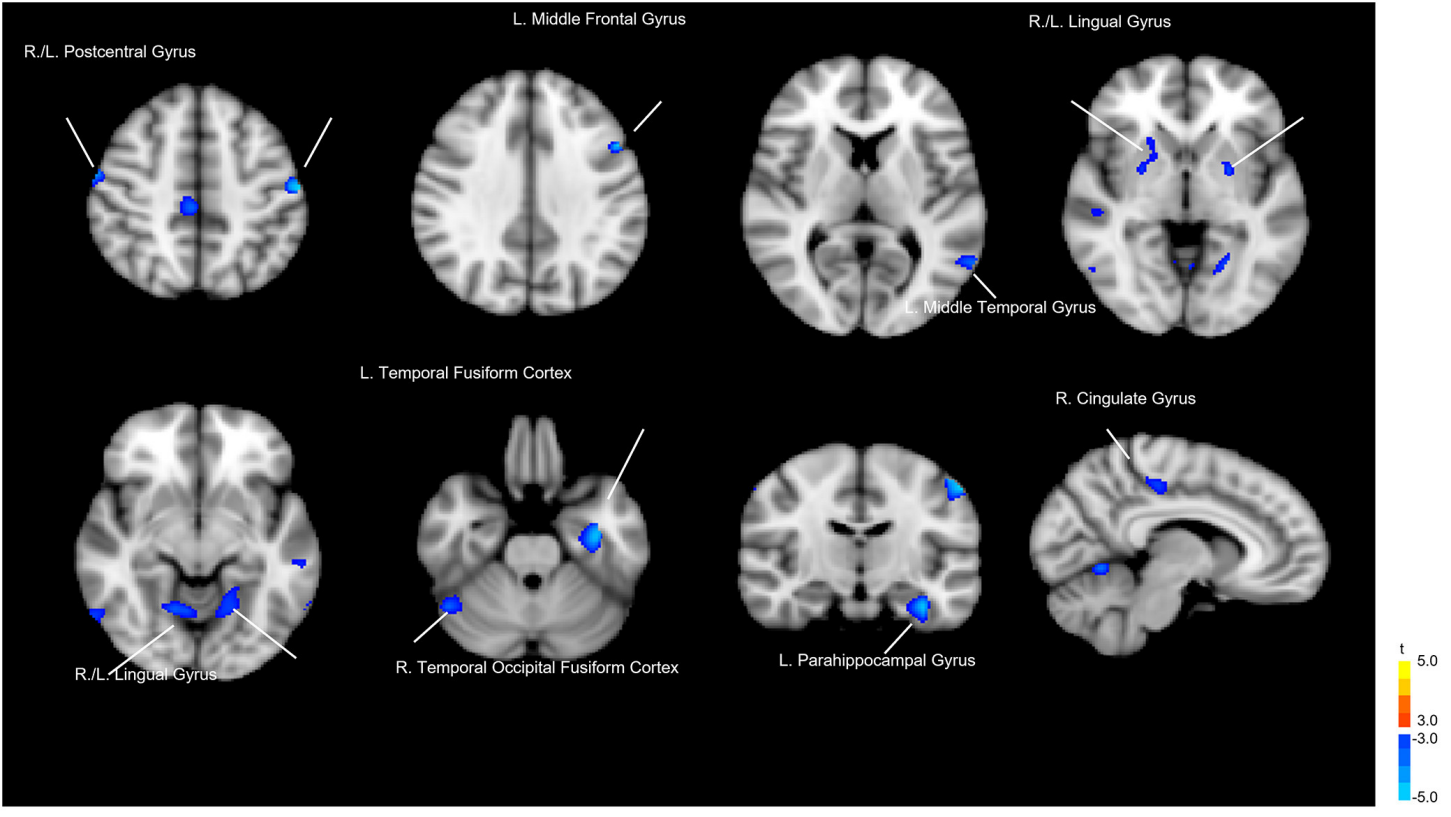
Gray matter volume differences between the patients with late-life depression (LLD) and healthy controls. Blue areas indicate brain regions where gray matter volume was significantly reduced.

Whole-brain voxelwise *t*-tests were used to detect differences in local GM volume between the 2 groups. To avoid possible edge effects around the margin between different tissue types, all voxels with a GM probability value < 0.2 (absolute threshold; range, 0–1) were excluded. The threshold was set at *p* < 0.05 (corrected for false discovery rate [FDR]) at the cluster level with a voxelwise *p* < 0.001 using a combined height and extent threshold technique based on 10,000 Monte Carlo simulations calculated through the Analysis of Functional NeuroImages (AFNI) program, 3dClustSim (the successor of AlphaSim; Cox, 1996; http://afni.nimh.nih.gov/pub/dist/doc/program_help/3dClustSim.html). Based on the results of the Monte Carlo simulation, we considered a minimum of 30 voxels as the threshold. The regional GM volumes were extracted for each participant from the significant clusters in the group comparison. Figure 1 and Table 1 show the results of the significant difference in GM volume between groups. Compared to the healthy control group, the LLD group had reduced GM volume in the left (L.) temporal fusiform cortex, L. lingual gyrus, L. postcentral gyrus, right (R.) lingual gyrus, R. temporal occipital fusiform cortex, R. postcentral gyrus, R. cerebellar crus II, R. cingulate gyrus, posterior division, L. cerebellar vermis VIIIa, R. cingulate gyrus, posterior division, L. cerebellar vermis VIIIa, L. middle temporal gyrus, L. parahippocampal gyrus, L. middle frontal gyrus, R. lateral occipital cortex, and L. putamen. Those areas were defined as regions of interest (ROIs), and then Spearman's rank order correlation analysis was used to measure the degree of association between these ROIs and the microbiota that characterize LLD (Please refer to the section below).

**Table 1:** Regional differences in gray matter volume between patients with late-life depression (LLD) and healthy controls (HC).

	**Cluster size**	**Coordinate**			**Harvard-Oxford cortical**
		**x**	**y**	**z**	**Structural atlas**
LLD > HC	-	-	-	-	
HC > LLD	459	−38	−9	−28	L. Temporal fusiform cortex
	329	−20	−48	−12	L. Lingual gyrus
	186	−57	−16	46	L. Postcentral gyrus
	136	6	−58	−4	R. Lingual gyrus
	108	46	−57	−24	R. Temporal occipital fusiform cortex
	104	57	−8	45	R. Postcentral gyrus
	91	48	−51	−46	R. Cerebellar crus II
	85	3	−30	48	R. Cingulate gyrus, posterior division
	75	0	−70	−46	L. Cerebellar vermis VIIIa
	68	−62	−58	8	L. Middle temporal gyrus
	60	−33	−32	−16	L. Parahippocampal gyrus
	49	−50	12	33	L. Middle frontal gyrus
	44	58	−63	−8	R. Lateral occipital cortex
	31	−42	−80	−38	L. Cerebellar crus II
	30	−24	−2	−2	L. Putamen

L, left; R, right.

#### Bioinformatics analysis of gut microbiota

We followed a standardized microbiome analysis pipeline including preprocessing, quality control, taxonomic classification and determination of microbiome diversity. In brief, after polymerase chain reaction (PCR) amplification and sequencing on the Illumina platform, raw fastq files were preprocessed in QIIME2 (Bolyen et al., 2019). Pair-end sequence primer adaptors were trimmed by cutadapt (Martin, 2011). (via q2-cutadapt). Then, sequences were quality filtered and denoised by the DADA2 algorithm (Callahan et al., 2016) (via q2-dada2) to identify amplicon sequence variants (ASVs) (Callahan et al., 2017). ASVs were aligned to construct a phylogenetic tree by mafft (Katoh and Standley, 2013) and fasttree2 (Price et al., 2010) (via q2-phylogeny). The alpha- (α; Shannon) (Shannon, 1948) and Faith's phylogenetic diversity [FPD] (Faith, 1992) and beta- (β; unweighted UniFrac distance) (Lozupone and Knight, 2005) diversity metrics and principal coordinate analysis (PCoA) were calculated from rarefied samples using q2-diversity. The statistical analysis and figure drawing of α-diversity metrics were implemented in the R environment by the ggplot2 (Wickham, 2009) and ggpubr (Kassambara, 2020) packages. The taxonomic classification was conducted by a naïve Bayes taxonomy classifier (via q2-feature-classifier classify-sklearn) 00 (Bokulich et al., 2018) through the reference sequence from 6 databases (MetaSquare, Silva, Greengenes, RDP, HOMD, and Ezbiocloud) (Yoon et al., 2017; Sierra et al., 2020). We also added the latest published novel species sequences to MetaSquare without redundancy. Based on MetaSquare, we had higher resolution in microbial taxonomy and fewer unclassified sequences.

#### Analysis to determine LLD-associated microbiota and correlations with brain structures

Linear discriminant analysis (LDA) effect size (LEfSe) (Segata et al., 2011) was conducted to examine which microbes most explained the difference between LLD patients and controls. Microbiota with LDA scores (log 10) > 2 were considered significantly different in abundance between the two groups. A Mantel test (Mantel, 1967) was conducted to test for correlations between variations in GM volume and the β-diversity distance matrix in the R environment *via* the vegan package. The Mantel test statistic was the Spearman correlation between matrices, and significance was calculated using a permutation test with 9999 permutations. Then, the GM regions with significant *p* values (< 0.05) were selected after the Mantel test to evaluate their correlations with the depression-related microbes identified from the LEfSe test using the Spearman method.

## Results

### Demographic characteristics

The mean age of adults in the present study was 65.2 ± 7.7 years old (ranging from 50–86 years). In depressed group, the mean duration of illness was 18.2 ± 15.7 months (ranging from 6–48 months), and 14 (43.8%) patients were under regular treatment with selective serotonin reuptake inhibitors, 9 (28.1%) with serotonin-norepinephrine reuptake inhibitors, and 9 (28.1%) with agomelatine. The LLD patients exhibited more depressive symptoms with higher HAMD scores (13.6 ± 7.3 vs. 1.5 ± 1.8, *p* < 0.001) and poorer sleep quality with higher PSQI scores (12.4 ± 4.6 vs. 6.7 ± 3.7, *p* < 0.001) than the controls. There was no significant difference in age, sex, BMI, history of constipation/diabetes, educational level, or dietary patterns between the LLD patients and controls. However, intake of dietary fibers was marginally increased in healthy controls than LLD patients (*p* = 0.07) (Table 2).

**Table 2:** Demographic and clinical data of the patients with late-life depression (LLD) and healthy controls (HCs).

**Subject characteristics**	**LLD** **(*****n** =* **36)**	**HC** **(*****n** =* **17)**	***p***
Sex (F/M)	28/8	9/8	0.11
Age (years)	65.6 ± 7.3	64.1 ± 7.9	0.53
Education (years)	9.7 ± 4.8	10.9 ± 5.2	0.42
BMI	23.4 ± 3.2	24.5 ± 3.2	0.23
HAMD	13.6 ± 7.3	1.6 ± 1.8	<0.001
MoCA	22.9 ± 5.2	24.8 ± 3.9	0.15
PSQI	12.4 ± 4.6	6.7 ± 3.7	<0.001
Constipation	3(8.3%)	1(5.9%)	0.712
Diabetes	2 (5.6%)	1 (5.9%)	1.00
Diet pattern			
Protein	147.9 ± 8.3	266.1 ± 446.2	0.29
Dairy product	9.3 ± 18.0	56.4 ± 171.5	0.28
Vegetable	13.9 ± 9.1	70.6 ± 180.9	0.21
Fruit	13.4 ± 27.1	26.9 ± 54.3	0.34
Sugar	22.6 ± 20.1	38.9 ± 73.5	0.38
Dietary fiber	23.0 ± 16.9	102.5 ± 240.6	0.07
Sweet beverages	4.2 ± 10.1	6.1 ± 8.6	0.51
Processed foods	39.0 ± 33.4	47.8 ± 38.0	0.41

M, male; F, female; BMI, body mass index; HAMD, Hamilton Depression Scale; MoCA, Montreal Cognitive Assessment; PSQI, Pittsburgh Sleep Quality Index.

### Differences in microbiota composition between LLD patients and controls

**Figure 2:**
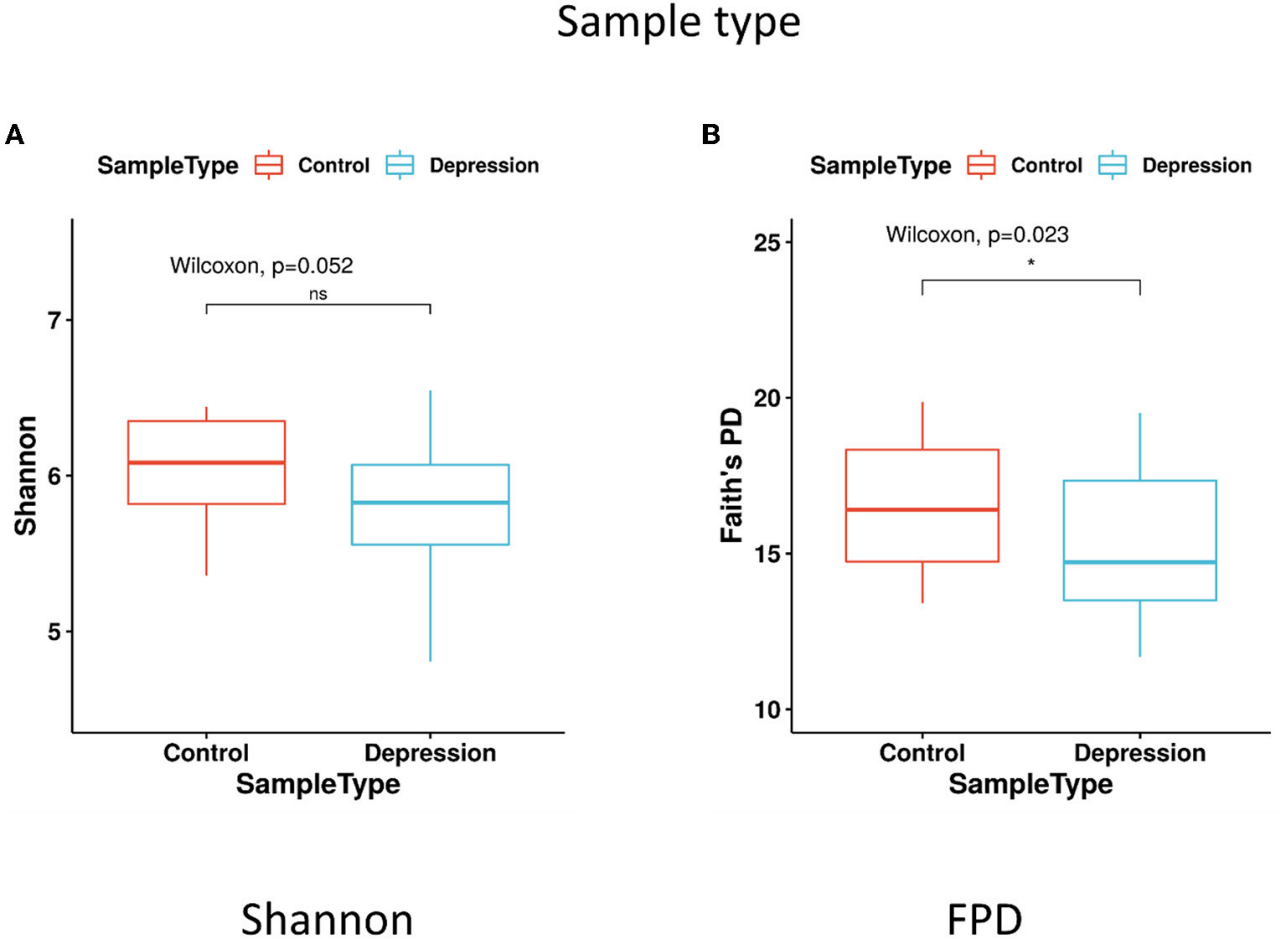
α-diversity in fecal samples shown as boxplots. α-diversity, measured by **(A)** Shannon diversity index and **(B)** Faith's phylogenetic diversity (FPD), is plotted for patients with late-life depression (LLD) (blue) and controls (red). The thick line inside the box represents the median, while the whiskers represent the lowest and highest values within the 1.5 interquartile range (IQR). The Wilcoxon rank-sum test shows that FPD is significantly decreased in depressed patients compared to controls. *p < 0.05 by Wilcoxon rank-sum test.

**Figure 3:**
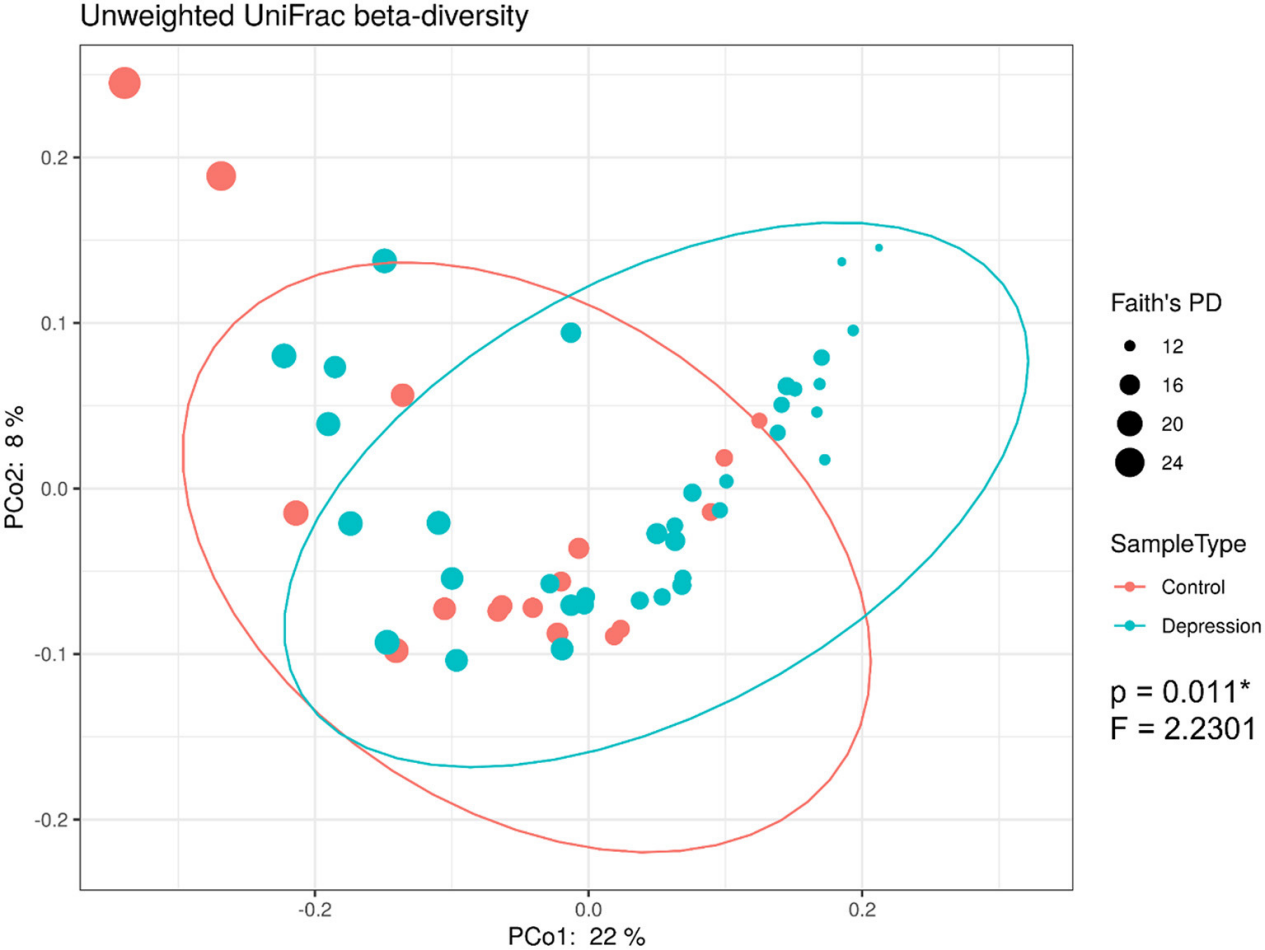
16S rRNA gene data revealed significant beta-diversity between the gut microbiota from late-life depression (LLD) patients and healthy controls. Principal coordinate analysis (PCoA) on the unweighted UniFrac distance matrix from the rarefied data was used to evaluate the presence of clusters or groupings based upon operational taxonomic unit (OTU)-level microbial features.

The within-sample (α) phylogenetic diversity analysis (by FPD) showed that the LLD group had significantly lower α-diversity than the control group (Figure 2). Using unweighted UniFrac analysis to detect the degree of microbial phylogenetic similarity (β-diversity), the three-dimensional plots of unweighted UniFrac analysis showed a significant difference in the gut microbial composition between LLD patients and healthy controls (Figure 3).

**Figure 4:**
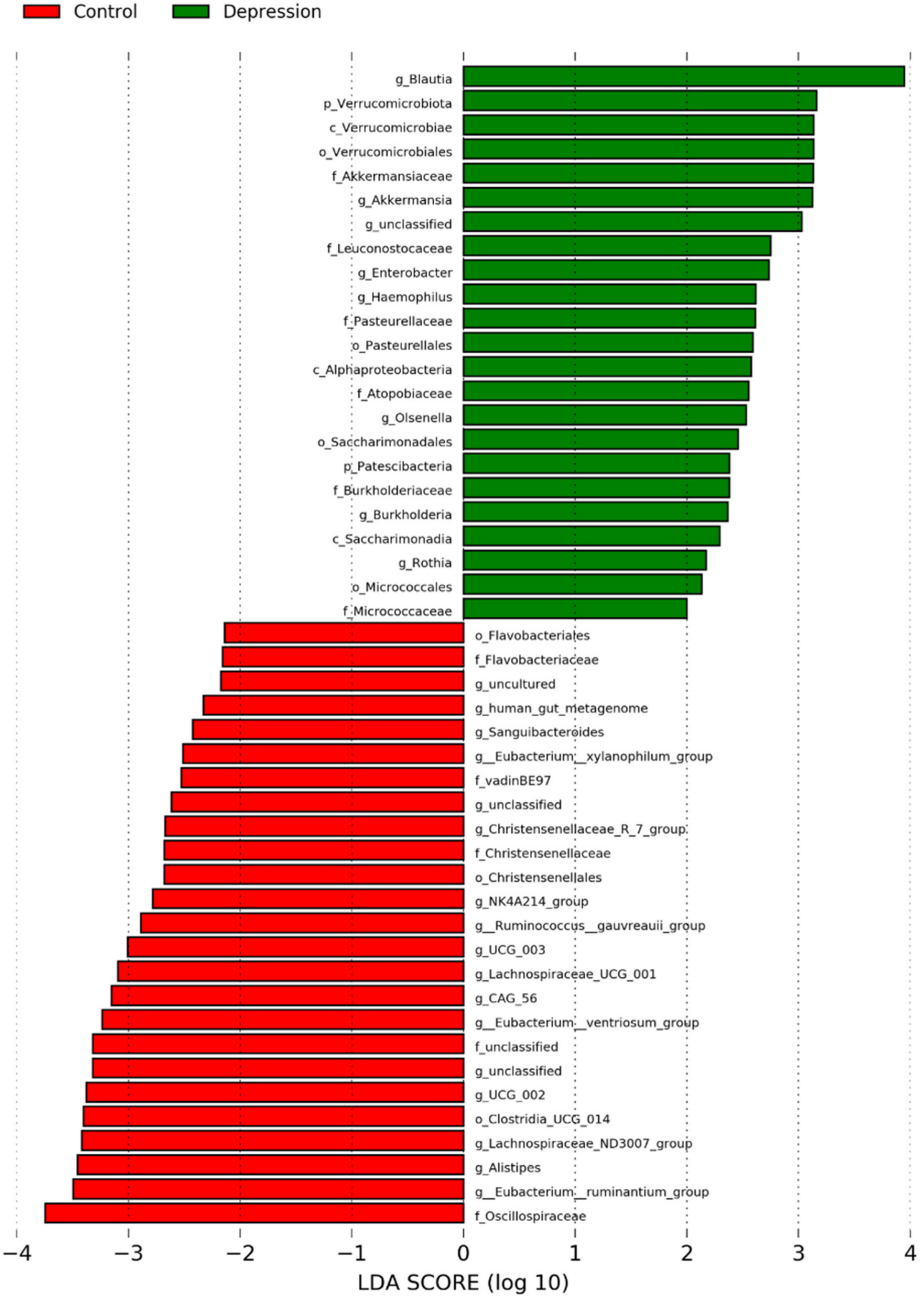
Abundances of bacterial taxa at the genus level in the late-life depression (LLD) patient and healthy control groups, as determined with the linear discriminant analysis (LDA) effect size (LEfSe) method. The taxonomic cladogram was generated based on the LEfSe and LDA scores. LLD-enriched taxa are indicated with a positive LDA score (green), and taxa enriched in controls have a negative score (red). Only taxa meeting the LDA significance threshold >2 are shown.

Analyses for each microbial taxon showed that the distribution of some microbiota targets at the phylum, class, order, family and genus levels was different between LLD patients and healthy controls (Figure 4). Our data revealed that several targets were more abundant in the LLD patients, including 2 phyla (*Verrucomicrobiota and Patescibacteria*), 3 classes (*Verrucomicrobiae, Alphaproteobacteria*, and *Saccharimonadia*), 4 orders (*Verrucomicrobiales, Pasterurellales, Saccharimonadales*, and *Micrococcales*), 6 families (*Akkermansiaceae, Burkholderiaceae, Pasteurellaceae, Micrococcaceae Leuconostocaceae* and *Atopobiaceae*) and 7 genera (*Eggerthella, Blautia, Olsenella, Haemophilus, Enterobacter, Burkholderia*, and *Rothia*). In addition, the healthy-enriched microbiota included 3 orders, 5 families, and 17 genera as shown in Figure 4.

### Associations between microbiota taxa differentiating groups and clinical indicators

**Figure 5:**
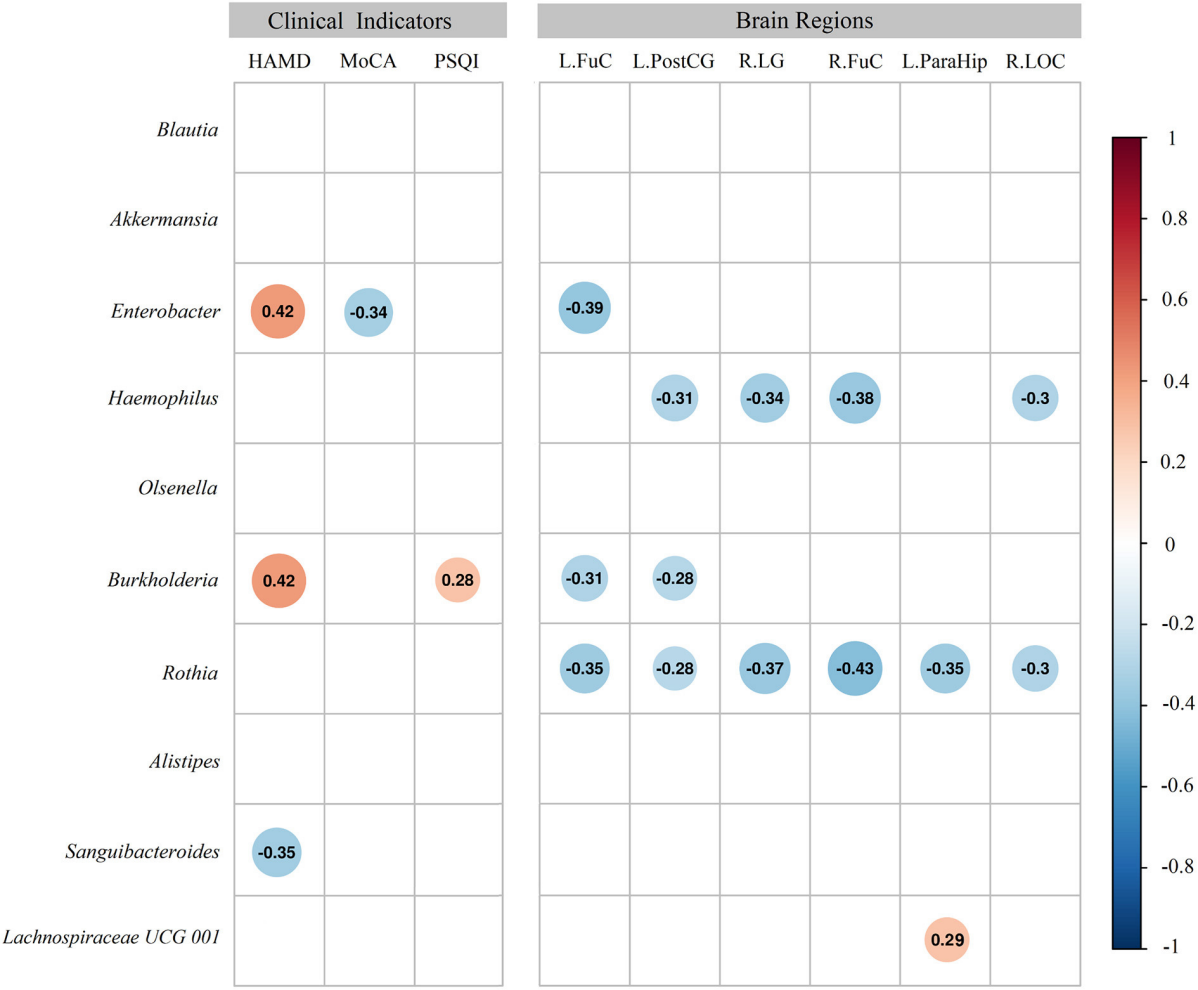
Heatmap of Spearman's rank correlation coefficients of the relative abundances of different gut microbiota at the genus level with clinical indices and regions with brain morphometric differences. Color intensity represents the magnitude of the correlation (blue circles = negative correlations; red circles = positive correlations).

The genera *Enterobacter* (*r* = 0.426, *p* = 0.002) and *Burkholderia* (*r* = 0.421, *p* = 0.002), among the richer genera in the LLD patients, showed positive correlations with HAMD scores, while the genus *Sanguibacteroides*, a richer genus in the healthy controls, showed a negative correlation with HAMD scores (*r* = −0.347, *p* = 0.011). Regarding cognitive function, the genus *Enterobacter* (*r* = −0.336, *p* = 0.014) also displayed a negative correlation with MoCA scores. Regarding sleep disturbance, the genus *Burkholderia* (*r* = 0.284, *p* = 0.039) showed a positive correlation with PSQI scores (Figure 5).

### Associations between microbiota taxa and regional brain structures

**Figure 6:**
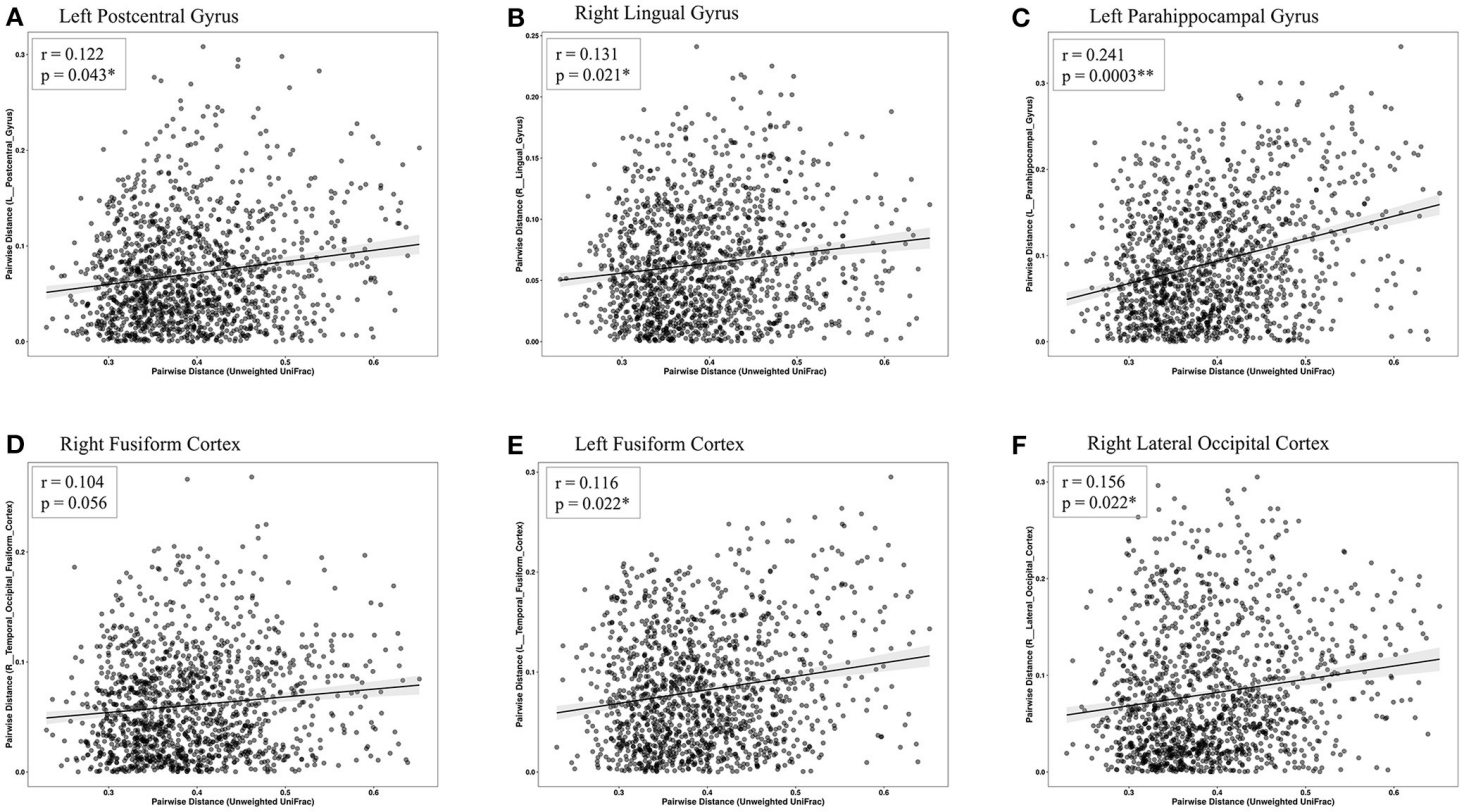
Scatterplots of Euclidean distance. The associations between fecal microbiome β-diversity (Euclidean distance centered log-transformed counts) and the gray matter distance separating pairs of late-life depression (LLD) patients or healthy controls in the **(A)** left postcentral gyrus, **(B)** right lingual gyrus, **(C)** left parahippocampal gyrus, **(D)** right fusiform cortex, **(E)** left fusiform cortex, and **(F)** right lateral occipital cortex. Each point represents a pair of LLD patients or healthy controls.

The Mantel test highlighted that Euclidean distance of the left temporal fusiform cortex, left postcentral gyrus, right lingual gyrus, right temporal occipital fusiform cortex, left parahippocampal gyrus, and right lateral occipital cortex were significantly correlated with the distances of the microbial community composition between participants (Table 3 and Figure 6). Subsequent analysis revealed that the genera *Enterobacter* (phylum *Proteobacteria), Rothia* (phylum *Actinobacteria), Haemophilus (*phylum *Proteobacteria)*, and *Burkholderia* (phylum *Proteobacteria)* were negatively associated with GM differences. Among the healthy-enriched genera, *Lachnospiraceae_UCG_001* was found to be positively correlated with GM volumes in left parahippocampal gyrus (*r* = 0.29, *p* = 0.03) (Figure 5).

**Table 3:** Summary results of Mantel tests showing *r* and *p* values of the matrix correlation between gray matter and β-diversity metrics.

**Gray matter**	***r***	***p***
L. Temporal fusiform cortex	0.116	0.0224
L. Postcentral gyrus	0.122	0.0434
R. Lingual gyrus	0.131	0.0213
R. Temporal occipital fusiform cortex	0.104	0.0569
L. Parahippocampal gyrus	0.241	0.0003
R. Lateral occipital cortex	0.156	0.0216

L, left; R, right.

## Discussion

This study characterized the gut microbiota signature in elderly MDD patients. We confirmed that the gut microbiota taxonomic composition and diversity were significantly different in LLD patients compared with healthy controls. We found that the genera *Enterobacter* and *Burkholderia* positively correlated with depressive symptoms and negatively correlated with brain structural signatures related to memory and emotional processing/cognition/regulation. We may be the first to demonstrate the linkage of the differences in microbiota taxa with depressive symptoms and reduced GM volumes in LLD patients when compared with controls.

### Diversities of gut microbiota in LLD patients and controls

Our study identified that LLD patients showed lower α-diversity than controls. Differences in α-diversity of the microbiota between depressed patients and controls has not been consistent in the literature. While higher α-diversity was found in adult patients with MDD than in controls in several studies (Kelly et al., 2016; Liu et al., 2016), one study found an opposite trend by using the Shannon index (Jiang et al., 2015). Other studies showed no differences in α-diversity between depressed patients and healthy controls (Naseribafrouei et al., 2014; Zheng et al., 2016; Lin et al., 2017; Chen et al., 2018a; Chung et al., 2019). The discrepancies among these studies contributing to the inconsistent findings are not clear, but they may be due to differences in patient populations (e.g., elderly patients in the current study) and statistical methods/parameters (Shannon index, Simpson index, phylogenetic diversity, total observed species, Chao 1, and FPD). Similarly, inconsistent results in previous studies have also been identified regarding β-diversity between depressed patients and controls. Seven studies found significant differences in β-diversity between depressed patients and controls by using PCoA analysis and weighted UniFrac (Kelly et al., 2016; Zheng et al., 2016; Lin et al., 2017; Chen et al., 2018a; Huang et al., 2018; Chung et al., 2019). Five studies found no differences in β-diversity in participants with a depressive disorder relative to controls (Jiang et al., 2015; Barandouzi et al., 2020). In the current study, we demonstrated a significant difference in the β-diversity of the gut microbiota between LLD patients and controls based on the three-dimensional plots of unweighted UniFrac analysis. Again, we suspected that the choice of clinical stratification criteria to enroll depressed patients as well as the analytic methods can be the factors responsible for the discrepant results.

In the present study, a number of microbiota targets at the phylum, class, order, family and genus levels showed different distributions between LLD patients and controls. Previous studies in adult Chinese individuals (mean age between 22 and 45 years old) consistently reported that *Actinobacteria* was more abundant in MDD patients than in controls (Jiang et al., 2015; Liu et al., 2016; Zheng et al., 2016; Lin et al., 2017; Chen et al., 2018a; Chung et al., 2019). At the phylum level, while we identified *Verrucomicrobiota* and *Patescibacteria* to be increased in the LLD group. At the family level, we identified several taxa that were more abundant in LLD patients. Some of them have been found to be linked to mental health, including depressive disorders, anxiety disorders, and personality traits. In patients with active generalized anxiety disorder, a higher relative proportion of *Burkholderiaceae* was reported (Chen et al., 2019), and a higher abundance of *Pasteurellaceae* was observed to be associated with greater pregnancy-related anxiety (Hu et al., 2019) and neuroticism personality traits in adults (Kim et al., 2018). Moreover, *Micrococcaceae* was shown to be more represented in untreated patients with MDD (Fontana et al., 2020). We demonstrated that *Leuconostocaceae* and *Atopobiaceae* at the family level were more abundant in the LLD patients, which had never been reported in the previous literature for mood disorders. Whether advanced age or other factors contribute to this discrepancy deserves further evaluation.

Our study showed that the LLD patients exhibited higher levels of the family *Akkermansiaceae* and genus *Akkermansia* than the controls. This finding is interesting since recent reports have shown reductions in the relative abundance of *Akkermansia* in socially defeated mice with anxiety- and depressive-like behavior (McGaughey et al., 2019). The neurobiological basis of LLD symptoms has been hypothesized to be associated with PD (Nishiwaki et al., 2020b). Furthermore, a longitudinal cohort study suggested that neurodegeneration and neuronal density in the mesolimbic dopamine system are related to LLD symptoms (Wilson et al., 2013). On the other hand, late-onset depression was observed with increased rates of clinical and imaging features that were associated with prodromal PD and dementia of Lewy body (Kazmi et al., 2021). Taken together, our findings may provide indirect evidence to support a link between LLD and parkinsonism-related pathophysiology. Previous studies have revealed that intestinal mucosal dysfunction characterized by an increased translocation of gram-negative bacteria (leaky gut) plays a role in the inflammatory pathophysiology of depression (Maes et al., 2012). *Akkermansia muciniphila* may degrade the mucus layer of the intestine (Derrien et al., 2004) and erode the colonic mucus layer in the absence of dietary fibers (Desai et al., 2016). The genus *Akkermansia* has been suggested to increase gut permeability and may enhance intestinal neural plexus exposure to oxidative stress and the inflammatory response, which would further facilitate the subsequent development of neuropsychiatric symptoms (Nishiwaki et al., 2020a). Increased abundance of *Akkermansia* genus, which is the most consistent finding in PD (Tan et al., 2022), were identified in our LLD patients. Though not reaching at significant levels, these LLD patients displayed a decreased fiber intake and increased rate of constipation, which phenomenon are also commonly displayed in PD. All these observations would further support our speculation regarding the potential association between LLD and PD. Despite of the facts, the interactions among *Akkermansia*, fiber intake and constipation in the PD pathogenesis are complex. For example, fiber intake would either increase or exert no effect on the abundance of genus *Akkermansia* (Verhoog et al., 2019; Rodríguez-Lara et al., 2022). Actually, the associations between the mentioned factors and PD development in the available human studies are largely correlative, which correlations may or may not be the causes leading to PD. The observed correlations may just reflect secondary or shared common and non-specific responses to PD, such as delayed gut transit, alterations in appetite, or even inflammation (Tan et al., 2022). Further large-scaled and longitudinal follow-up studies are necessary to elucidate the issue.

Besides *Akkermansia*, we also found genera *Burkholderia* and *Enterobacter* are richer in the LLD patients, which bacteria are also linked with the PD development. For example, the appendix of PD patients displayed a prominent increase in *Burkholderiales*, which is reported to infect the brain *via* olfactory system (Walkden et al., 2020; Li et al., 2021). Furthermore, *Burkholderiales* would produce kynurenine and quinolinate, which are proinflammatory metabolites associated with symptom severity in PD (Kaur et al., 2019; Heilman et al., 2020). PD patients also displayed higher levels of Enterobacteriaceae, which were in association with the degree of gait and postural instability (Scheperjans et al., 2015). The 3 genera *Akkermansia, Burkholderia* and *Enterobacter* enriched in our LLD patients are all PD-related. Future longitudinal follow-up study can be interesting to evaluate the potential PD development among the LLD patients enriched with *Akkermansia, Burkholderia* and *Enterobacter*.

At the genus level, we found some results consistent with similar trends across studies, such as *Eggerthella* (Kelly et al., 2016; Chen et al., 2018a), *Blautia* (Jiang et al., 2015), *Olsenella* (Chen et al., 2018a), and *Alistipes* (Naseribafrouei et al., 2014; Nishiwaki et al., 2020a), while some other reported microbiota targets showed different directions in the associations with depressive disorders, including *Haemophilus* (Nishiwaki et al., 2020a) and *Alistipes* (Zheng et al., 2016). The reasons for the discrepancies are unknown, but it might have been caused by diverse characteristics in samples, such as age, depressive severity, disease phenotype, comorbid conditions, diet, and other unmeasured confounding factors among patients, and by different study designs.

### Correlations of microbiota abundance and clinical characteristics

We found significant correlations between the abundance of some gut microbiota and clinical characteristics of LLD. The richness of the genus *Enterobacter* was related to a higher severity of depressive symptoms and poor cognitive performance in the present study, and a higher abundance of the family *Enterobacteriaceae* was consistently found in patients with either MDD or bipolar disorder (Jiang et al., 2015; Guo et al., 2018). Since cognitive impairment is frequently observed in patients with LLD and is also associated with a greater likelihood of developing all-cause dementia (Diniz et al., 2013), it is worth exploring the links between the microbiome and mood/cognition in the elderly population in future research. Moreover, we found that the richness of the genus *Burkholderia* was associated with 2 core symptoms of depressive disorder, i.e., higher severity of depression and poor sleep quality. *Burkholderia* is involved in the metabolism of tryptophan, a precursor for serotonin in mood regulation (O'Mahony et al., 2015), which may contribute to depression through the microbiota-gut-brain axis pathway. Previous studies have shown a trend association in the genus *Blautia* and a significant association between depression scores and the family *Peptostreptococcaceae*, genus *Prevotella*, genus *Klebsiella*, genus *Sutterella* and genus *Eggerthella* (Lin et al., 2017; Chung et al., 2019). In addition, the abundance of the genus *Faecalibacterium* was found to be either negatively (Jiang et al., 2015) or positively (Chen et al., 2018b) associated with depressive symptoms. The reasons for the widely inconsistent results across studies remain unclear. It is mandatory to identify aspects of the gut microbiome and other biological factors/metabolites associated with MDD to delineate MDD heterogeneity.

### Correlations of gut microbiota with regional GM volumes

To the best of our knowledge, this is the first study to reveal significant correlations between regional GM volumes and gut microbiota in LLD patients when compared with healthy controls. Specific microbial taxa found to characterize LLD in the present study showed negative correlations with GM volumes in several cortical and subcortical brain regions that are mainly involved in memory, somatosensory integration, and emotion processing, recognition, and regulation (Kawasaki et al., 2012; Saarimaki et al., 2016).

Previous studies have already demonstrated that MDD patients show both cortical and subcortical structural differences, such as reduced GM volumes in some of the same brain regions mentioned above (Reinecke et al., 2015). Our findings provide further evidence to support the link between brain structural changes and gut microbiota, which would strengthen the role of the microbiome-gut-brain axis in the pathogenesis of mood disorders. We identified that reduced GM volume in the left postcentral gyrus was linked with a higher abundance of the genera *Haemophilus, Rothia*, and *Burkholderia*. Notably, a smaller left postcentral gyrus has been suggested as an imaging marker for higher severity of depression among adult MDD patients by a large working group composed of 20 international cohorts worldwide (Schmaal et al., 2017). Reduced volume in the left temporal fusiform cortex was associated with a higher abundance of the genera *Rothia, Enterobacter*, and *Burkholderia*. Both the temporal fusiform cortex region and postcentral gyrus (also belonging to the somatosensory cortex) are crucial regions for emotional processing/recognition and multisensory integration (Kawasaki et al., 2012; Saarimaki et al., 2016). In addition, reduced volumes in the right lingual gyrus, right temporal occipital fusiform cortex, and right lateral occipital cortex were correlated with the abundance of the same bacterial genera (genus *Haemophilus* and genus *Rothia*). The lingual gyrus, parahippocampal gyrus and lateral occipital cortex are involved in facial emotional expression and emotion regulation (Reinecke et al., 2015). Interestingly, we found that the abundance of the genus *Rothia* was negatively correlated with most regions showing GM volume reduction. *Rothia* species are gram-positive bacteria that normally inhabit the human oral cavity and respiratory tract and are usually associated with dental caries, septicemia, and even central nervous system infections (Goldman et al., 1998). Regarding mental disorders, *Rothia* species were found to be statistically more prevalent in children with autism spectrum disorder than in healthy controls (Forsyth et al., 2020). Furthermore, microbiota from the oral cavity can affect neurocognition through proinflammatory cytokines (Ranjan et al., 2018). We then speculated that *Rothia* species might be involved in systemic inflammation and the immune response in the pathogenesis of brain disorders.

A recent study by enrolling 16 Caucasian LLD patients without controls showed that the genera *Lachnospiraceae_NK4A136, Oscillibacter*, and unclassified *Ruminococcaceae* were positively associated with the GM volume in hippocampus and amygdala (Lee et al., 2022). We identified genus *Ruminococcus*, genus *Lachnospiraceae* and family *Oscillospiraceae*, were significantly enriched in healthy controls than in LLD. Among these bacteria, we found the genus *Lachnospiraceae*_UCG_001 is positively correlated with gray matter volumes (r = 0.29, *p* = 0.03) in left parahippocampal gyrus. However, no significant association could be found between genus *Ruminococcus*, family *Oscillospiraceae* and gray matter volumes. Such results may suggest that the changes of these taxa may contribute to the pathophysiology of LLD, partly through the alterations in GM volume. Nevertheless, the differences in age, patient numbers, ethnicity, and study design (presence of control group) may contribute to the discrepancies between the two studies. Further large-scaled studies are mandatory to clarify this issue.

In the current study, we found that *Enterobacter* and *Burkholderia* were the 2 unique genera correlated with both clinical characteristics of depression and reduced regional brain volumes. This finding may imply the potential role of these 2 specific microbes as precise targets for future microbiota-directed interventional therapies.

The biological mechanisms underlying the observed relationships between gut microbiota and brain structural differences remain to be determined. The interactions between gut microbiota and the brain may be meditated by neurodevelopment, neuroendocrine systems, microbial metabolites, and immune-related molecular pathways resulting in the observed phenomena (Diaz Heijtz et al., 2011). It has been suggested that neurotoxic and inflammatory metabolites generated by altered gut microbiota reach the brain, resulting in neuroplastic changes in brain structures in somatosensory brain regions (Labus et al., 2017). Recent structural brain imaging studies have also provided evidence that support an interaction between gut microbiota and the brain through the mentioned pathway. For example, in healthy women, patients with irritable bowel syndrome, or elderly patients with cirrhosis, there were significant correlations between increased metabolites, pro-inflammatory cytokines/endotoxin, and structural differences in brain regions (Labus et al., 2017; Liu et al., 2019).

There are several limitations in this study. First, the sample size of the study was moderate and inadequate for subgroup analyses. A sex effect related to how the gut microbiota contributed to the observed results cannot be excluded since both sexes were enrolled. Fiber intake was marginally increased in our control subjects. Dietary fibers would interact with gut microbiota, leading to the production of key metabolites such as short-chain fatty acids, and impacts gut microbial ecology (Makki et al., 2018). Therefore, the difference in fiber intake between the groups may be resulted from the differences in food intake or the depression *per se*. Additionally, gut microbial metabolites were not measured to explore potential mediators during the process of depression development and GM differences. Furthermore, all the identified correlations were cross sectional, and no conclusion relating to causality can be made from the current findings. The observed brain volume differences could be consequences of altered signaling to the brain through microbiota, the depressive illness itself, or both (Liu et al., 2019). Finally, the depressed patients in this study were mainly elderly followed up in outpatient clinics and without regular usage of laxatives. Thus, the current findings may not be applicable to depressed patients at a younger age, those under hospitalization, or with comorbidity with constipation. Furthermore, we did not enquire the exact frequency of bowel habit per week. And chronic constipation is more commonly identified in depressed individuals than non-depressed individuals (Ballou et al., 2019). Also, defecating frequency could impact the richness and composition of gut microbiota (Kwon et al., 2019). Therefore, future studies to investigate interactions among the stool frequency, depression, and gut microbiota are mandatory.

## Conclusions

In summary, this is, to our knowledge, the first report showing an association of gut microbial composition and regional brain GM volumes in elderly adults with MDD. The presence of the genera *Enterobacter* and *Burkholderia* was significantly correlated with depressive symptoms and reduced GM volume in regions associated with memory, somatosensory integration, and emotional processing. Nevertheless, future research is warranted to identify heterogeneous phenotypes of depression based not only on gut microbiota but also on metabolomics and brain signatures. It is crucial to obtain more robust features serving as reliable biomarkers to apply precise microbial interventions to reach adequate management for mental health in the elderly population.

## Data availability statement

The original contributions presented in the study are included in the article/supplementary files, further inquiries can be directed to the corresponding author/s.

## Ethics statement

The studies involving human participants were reviewed and approved by Taipei Veternas General Hospital Institutional Review Board. The patients/participants provided their written informed consent to participate in this study.

## Author contributions

C-FT, Y-PW, P-CT, C-YL, and C-LL: general design of the trial. C-FT, Y-PW, P-YL, P-SW, and C-LL: data acquisition. C-FT, Y-PW, C-HC, Y-BL, P-CT, C-YL, and C-LL: data analysis. C-FT, Y-PW, P-CT, C-YL, and C-LL: interpretation of the data. C-FT, Y-PW, C-HC, and P-CT: drafting of the manuscript. C-YL and C-LL: critical revision of the manuscript. All authors contributed to the article and approved the submitted version.

## Funding

This study was supported by grants from the Taipei Veterans General Hospital (V105C-201, V107C-153, V109C-136, V110C-119, VTA106-V1-6-1, VTA107-V1-9-1, and VTA109-V1-5-1) and the Ministry of Science and Technology, Taiwan (MOST 104-2314-B-075-040).

## Conflict of interest

The authors declare that the research was conducted in the absence of any commercial or financial relationships that could be construed as a potential conflict of interest.

## Publisher's note

All claims expressed in this article are solely those of the authors and do not necessarily represent those of their affiliated organizations, or those of the publisher, the editors and the reviewers. Any product that may be evaluated in this article, or claim that may be made by its manufacturer, is not guaranteed or endorsed by the publisher.

## References

[B1] Alexopoulos G. S. (2019). Mechanisms and treatment of late-life depression. Transl. Psychiat. 9, 188. 10.1038/s41398-019-0514-6 31383842 PMC6683149

[B2] American Psychiatric Association and American Psychiatric Association, Task Force on DSM-IV (2000). Diagnostic and Statistical Manual of Mental Disorders: DSM-IV-TR. Washington, DC: American Psychiatric Association.

[B3] Ballou S. Katon J. Singh P. Rangan V. Lee H. N. McMahon C.. (2019). Chronic diarrhea and constipation are more common in depressed individuals. Clin. Gastroenterol. Hepatol. 17, 2696–2703. 10.1016/j.cgh.2019.03.046 30954714 PMC6776710

[B4] Barandouzi Z. A. Starkweather A. R. Henderson W. A. Gyamfi A. Cong X. S. (2020). Altered composition of gut microbiota in depression: a systematic review. Front. Psychiatry 11, 541. 10.3389/fpsyt.2020.00541 32587537 PMC7299157

[B5] Bokulich N. A. Kaehler B. D. Rideout J. R. Dillon M. Bolyen E. Knight R.. (2018). Optimizing taxonomic classification of marker-gene amplicon sequences with QIIME 2's q2-feature-classifier plugin. Microbiome 6, 90. 10.1186/s40168-018-0470-z 29773078 PMC5956843

[B6] Bolyen E. Rideout J. R. Dillon M. R. Bokulich N. A. Abnet C. C. Al-Ghalith G. A.. (2019). Reproducible, interactive, scalable and extensible microbiome data science using QIIME 2. Nat. Biotechnol. 37, 852–857. 10.1038/s41587-019-0209-9 31341288 PMC7015180

[B7] Buysse D. J. Reynolds C. F. 3rd Monk T. H. Berman S. R. Kupfer D. J. (1989). The Pittsburgh Sleep Quality Index: a new instrument for psychiatric practice and research. Psychiatry Res. 28, 193–213. 10.1016/0165-1781(89)90047-4 2748771

[B8] Callahan B. J. McMurdie P. J. Holmes S. P. (2017). Exact sequence variants should replace operational taxonomic units in marker-gene data analysis. ISME J. 11, 2639–2643. 10.1038/ismej.2017.119 28731476 PMC5702726

[B9] Callahan B. J. McMurdie P. J. Rosen M. J. Han A. W. Johnson A. J. A. Holmes S. P. (2016). DADA2: High-resolution sample inference from Illumina amplicon data. Nat. Methods 13, 581–583. 10.1038/nmeth.3869 27214047 PMC4927377

[B10] Chen J. J. Zheng P. Liu Y. Y. Zhong X. G. Wang H. Y. Guo Y. J.. (2018a). Sex differences in gut microbiota in patients with major depressive disorder. Neuropsychiatr. Dis. Treat. 14, 647–655. 10.2147/NDT.S159322 29520144 PMC5833751

[B11] Chen Y. H. Bai J. Wu D. Yu S. F. Qiang X. L. Bai H.. (2019). Association between fecal microbiota and generalized anxiety disorder: severity and early treatment response. J. Affect. Disord. 259, 56–66. 10.1016/j.jad.2019.08.014 31437702

[B12] Chen Z. Li J. Gui S. Zhou C. Chen J. Yang C.. (2018b). Comparative metaproteomics analysis shows altered fecal microbiota signatures in patients with major depressive disorder. Neuroreport 29, 417–425. 10.1097/WNR.0000000000000985 29432299

[B13] Chung Y. E. Chen H. C. Chou H. L. Chen I. M. Lee M. S. Chuang L. C.. (2019). Exploration of microbiota targets for major depressive disorder and mood related traits. J. Psychiatr. Res. 111, 74–82. 10.1016/j.jpsychires.2019.01.016 30685565

[B14] Cox R. W. (1996). AFNI: Software for analysis and visualization of functional magnetic resonance neuroimages. Comput. Biomed. Res. Int. J. 29, 162–173. 10.1006/cbmr.1996.0014 8812068

[B15] Derrien M. Vaughan E. E. Plugge C. M. de Vos W. M. (2004). Akkermansia muciniphila gen. nov., sp. nov., a human intestinal mucin-degrading bacterium. Int. J. Syst. Evol. Microbiol. 54, 1469–1476. 10.1099/ijs.0.02873-0 15388697

[B16] Desai M. S. Seekatz A. M. Koropatkin N. M. Kamada N. Hickey C. A. Wolter M.. (2016). A dietary fiber-deprived gut microbiota degrades the colonic mucus barrier and enhances pathogen susceptibility. Cell 167, 1339–1353 e1321. 10.1016/j.cell.2016.10.043 27863247 PMC5131798

[B17] Diaz Heijtz R. Wang S. Anuar F. Qian Y. Bjorkholm B. Samuelsson A.. (2011). Normal gut microbiota modulates brain development and behavior. Proc. Natl. Acad. Sci. U S A. 108, 3047–3052. 10.1073/pnas.1010529108 21282636 PMC3041077

[B18] Diniz B. S. Butters M. A. Albert S. M. Dew M. A. Reynolds C. F. 3rd (2013). Late-life depression and risk of vascular dementia and Alzheimer's disease: systematic review and meta-analysis of community-based cohort studies. Br. J. Psychiat. 202, 329–335. 10.1192/bjp.bp.112.118307 23637108 PMC3640214

[B19] Disease G. B. D. Injury I. Prevalence C. (2018). Global, regional, and national incidence, prevalence, and years lived with disability for 354 diseases and injuries for 195 countries and territories, 1990-2017: a systematic analysis for the Global Burden of Disease Study 2017. Lancet 392, 1789–1858. 10.1016/S0140-6736(18)32279-7 30496104 PMC6227754

[B20] Dupont W. D. Plummer W. D. Jr. (1990). Power and sample size calculations. A review and computer program. Control Clin. Trials. 11, 116–128. 10.1016/0197-2456(90)90005-M 2161310

[B21] Faith D. P. (1992). Conservation evaluation and phylogenetic diversity. Biol. Conserv. 61, 1–10. 10.1016/0006-3207(92)91201-3

[B22] Fontana A. Manchia M. Panebianco C. Paribello P. Arzedi C. Cossu E.. (2020). Exploring the role of gut microbiota in major depressive disorder and in treatment resistance to antidepressants. Biomedicines 8, 311. 10.3390/biomedicines8090311 32867257 PMC7554953

[B23] Forsyth A. Raslan K. Lyashenko C. Bona S. Snow M. Khor B.. (2020). Children with autism spectrum disorder: Pilot studies examining the salivary microbiome and implications for gut metabolism and social behavior. Human Microb. J. 15, 100066. 10.1016/j.humic.2019.100066

[B24] Goldman M. Chaudhary U. B. Greist A. Fausel C. A. (1998). Central nervous system infections due to Stomatococcus mucilaginosus in immunocompromised hosts. Clin. Infect. Dis. 27, 1241–1246. 10.1086/515001 9827277

[B25] Guo L. Ji C. Ma Q. Fan Y. Feng J. Chen C.. (2018). The diversity and the abundance of gut microbiome in patients with bipolar disorder. Chin. J. Psychiatry.51:98–104.

[B26] Hamilton M. (1960). A rating scale for depression. J. Neurol. Neurosurg. Psychiat. 23, 56–62. 10.1136/jnnp.23.1.56 14399272 PMC495331

[B27] Hasler G. (2010). Pathophysiology of depression: do we have any solid evidence of interest to clinicians? World Psychiatry 9, 155–161. 10.1002/j.2051-5545.2010.tb00298.x 20975857 PMC2950973

[B28] Heilman P. L. Wang E. W. Lewis M. M. Krzyzanowski S. Capan C. D. Burmeister A. R.. (2020). Tryptophan metabolites are associated with symptoms and nigral pathology in Parkinson's disease. Mov. Disord. 35, 2028–2037. 10.1002/mds.28202 32710594 PMC7754343

[B29] Hu J. Ly J. Zhang W. Huang Y. Glover V. Peter I.. (2019). Microbiota of newborn meconium is associated with maternal anxiety experienced during pregnancy. Dev. Psychobiol. 61, 640–649. 10.1002/dev.21837 30908632 PMC6588465

[B30] Huang Y. Shi X. Li Z. Shen Y. Shi X. Wang L.. (2018). Possible association of Firmicutes in the gut microbiota of patients with major depressive disorder. Neuropsychiatr. Dis. Treat 14, 3329–3337. 10.2147/NDT.S188340 30584306 PMC6284853

[B31] Huang Y. C. Lee M. S. Pan W. H. Wahlqvist M. L. (2011). Validation of a simplified food frequency questionnaire as used in the Nutrition and Health Survey in Taiwan (NAHSIT) for the elderly. Asia Pac. J. Clin. Nutr. 20, 134–140. 21393121

[B32] Jiang H. Ling Z. Zhang Y. Mao H. Ma Z. Yin Y.. (2015). Altered fecal microbiota composition in patients with major depressive disorder. Brain Behav. Immun. 48, 186–194. 10.1016/j.bbi.2015.03.016 25882912

[B33] Kano M. Dupont P. Aziz Q. Fukudo S. (2018). Understanding neurogastroenterology from neuroimaging perspective: a comprehensive review of functional and structural brain imaging in functional gastrointestinal disorders. J. Neurogastroenterol. Motil. 24, 512–527. 10.5056/jnm18072 30041284 PMC6175554

[B34] Kassambara A. (2020). “*ggpubr: 'ggplot2' Based Publication Ready Plots”*.

[B35] Katoh K. Standley D. M. (2013). MAFFT multiple sequence alignment software version 7: improvements in performance and usability. Mol. Biol. Evol. 30, 772–780. 10.1093/molbev/mst010 23329690 PMC3603318

[B36] Kaur H. Bose C. Mande S. S. (2019). Tryptophan metabolism by gut microbiome and gut-brain-axis: an in silico analysis. Front. Neurosci. 13, 1365. 10.3389/fnins.2019.01365 31920519 PMC6930238

[B37] Kawasaki H. Tsuchiya N. Kovach C. K. Nourski K. V. Oya H. Howard M. A.. (2012). Processing of facial emotion in the human fusiform gyrus. J. Cogn. Neurosci. 24, 1358–1370. 10.1162/jocn_a_00175 22185494 PMC3566877

[B38] Kazmi H. Walker Z. Booij J. Khan F. Shah S. Sudre C. H.. (2021). Late onset depression: dopaminergic deficit and clinical features of prodromal Parkinson's disease: a cross-sectional study. J. Neurol. Neurosurg. Psychiatry 92, 158–164. 10.1136/jnnp-2020-324266 33268471 PMC7841491

[B39] Kelly J. R. Borre Y. C O.B. Patterson E. El Aidy S. Deane J.. (2016). Transferring the blues: Depression-associated gut microbiota induces neurobehavioural changes in the rat. J. Psychiatr. Res. 82, 109–118. 10.1016/j.jpsychires.2016.07.019 27491067

[B40] Kim H. N. Yun Y. Ryu S. Chang Y. Kwon M. J. Cho J.. (2018). Correlation between gut microbiota and personality in adults: a cross-sectional study. Brain Behav. Immun. 69, 374–385. 10.1016/j.bbi.2017.12.012 29278751

[B41] Kwon H. J. Lim J. H. Kang D. Lim S. Park S. J. Kim J. H. (2019). Is stool frequency associated with the richness and community composition of gut microbiota? Intest. Res. 17, 419–426. 10.5217/ir.2018.00149 30704159 PMC6667361

[B42] Labus J. S. Hollister E. B. Jacobs J. Kirbach K. Oezguen N. Gupta A.. (2017). Differences in gut microbial composition correlate with regional brain volumes in irritable bowel syndrome. Microbiome 5, 49. 10.1186/s40168-017-0260-z 28457228 PMC5410709

[B43] Lee S. M. Milillo M. M. Krause-Sorio B. Siddarth P. Kilpatrick L. Narr K. L.. (2022). Gut microbiome diversity and abundance correlate with gray matter volume (GMV) in older adults with depression. Int. J. Environ. Res. Public Health. 19, 2405. 10.3390/ijerph19042405 35206594 PMC8872347

[B44] Li P. Killinger B. A. Ensink E. Beddows I. Yilmaz A. Lubben N.. (2021). Gut microbiota dysbiosis is associated with elevated bile acids in Parkinson's disease. Metabolites 11, 29. 10.3390/metabo11010029 33406628 PMC7823437

[B45] Li Q. Zhao Y. Chen Z. Long J. Dai J. Huang X.. (2020). Meta-analysis of cortical thickness abnormalities in medication-free patients with major depressive disorder. Neuropsychopharmacology 45, 703–712. 10.1038/s41386-019-0563-9 31694045 PMC7021694

[B46] Li S. W. Watanabe K. Hsu C. C. Chao S. H. Yang Z. H. Lin Y. J.. (2017). Bacterial composition and diversity in breast milk samples from mothers living in Taiwan and Mainland China. Front. Microbiol. 8, 965. 10.3389/fmicb.2017.00965 28611760 PMC5447776

[B47] Liang S. Wu X. Hu X. Wang T. Jin F. (2018). Recognizing depression from the microbiota gut brain axis. Int. J. Mol. Sci. 19, 1592. 10.3390/ijms19061592 29843470 PMC6032096

[B48] Lin P. Ding B. Feng C. Yin S. Zhang T. Qi X.. (2017). Prevotella and Klebsiella proportions in fecal microbial communities are potential characteristic parameters for patients with major depressive disorder. J. Affect. Disord. 207, 300–304. 10.1016/j.jad.2016.09.051 27741466

[B49] Liu P. Peng G. Zhang N. Wang B. Luo B. (2019). Crosstalk between the gut microbiota and the brain: an update on neuroimaging findings. Front. Neurol. 10, 883. 10.3389/fneur.2019.00883 31456743 PMC6700295

[B50] Liu Y. Zhang L. Wang X. Wang Z. Zhang J. Jiang R.. (2016). Similar fecal microbiota signatures in patients with diarrhea-predominant irritable bowel syndrome and patients with depression. Clin. Gastroenterol. Hepatol. 14, 1602–1611 e1605. 10.1016/j.cgh.2016.05.033 27266978

[B51] Lozupone C. Knight R. (2005). UniFrac: a new phylogenetic method for comparing microbial communities. Appl. Environ. Microbiol. 71, 8228–8235. 10.1128/AEM.71.12.8228-8235.2005 16332807 PMC1317376

[B52] Maes M. Kubera M. Leunis J. C. Berk M. (2012). Increased IgA and IgM responses against gut commensals in chronic depression: further evidence for increased bacterial translocation or leaky gut. J. Affect. Disord. 141, 55–62. 10.1016/j.jad.2012.02.023 22410503

[B53] Makki K. Deehan E. C. Walter J. Bäckhed F. (2018). The impact of dietary fiber on gut microbiota in host health and disease. Cell Host Microbe 23, 705–715. 10.1016/j.chom.2018.05.012 29902436

[B54] Mantel N. (1967). The detection of disease clustering and a generalized regression approach. Cancer Res. 27, 209–220. 6018555

[B55] Martin C. R. Osadchiy V. Kalani A. Mayer E. A. (2018). The brain-gut-microbiome axis. Cell Mol. Gastroenterol. Hepatol. 6, 133–148. 10.1016/j.jcmgh.2018.04.003 30023410 PMC6047317

[B56] Martin M. (2011). Cutadapt removes adapter sequences from high-throughput sequencing reads. EMBnet. J. 17, 3. 10.14806/ej.17.1.200

[B57] McGaughey K. D. Yilmaz-Swenson T. Elsayed N. M. Cruz D. A. Rodriguiz R. M. Kritzer M. D.. (2019). Relative abundance of Akkermansia spp. and other bacterial phylotypes correlates with anxiety- and depressive-like behavior following social defeat in mice. Sci. Rep. 9, 3281. 10.1038/s41598-019-40140-5 30824791 PMC6397238

[B58] Morais L. H. Schreiber H. L. t Mazmanian S. K. (2021). The gut microbiota-brain axis in behaviour and brain disorders. Nat. Rev. Microbiol. 19, 241–255. 10.1038/s41579-020-00460-0 33093662

[B59] Naseribafrouei A. Hestad K. Avershina E. Sekelja M. Linlokken A. Wilson R.. (2014). Correlation between the human fecal microbiota and depression. Neurogastroenterol. Motil. 26, 1155–1162. 10.1111/nmo.12378 24888394

[B60] Nishiwaki H. Hamaguchi T. Ito M. Ishida T. Maeda T. Kashihara K.. (2020a). Short-chain fatty acid-producing gut microbiota is decreased in parkinson's disease but not in rapid-eye-movement sleep behavior disorder. mSystems 5, e00797-20. 10.1128/mSystems.00797-20 33293403 PMC7771407

[B61] Nishiwaki H. Ito M. Ishida T. Hamaguchi T. Maeda T. Kashihara K.. (2020b). Meta-analysis of gut dysbiosis in Parkinson's disease. Mov. Disord. 35, 1626–1635. 10.1002/mds.28119 32557853

[B62] O'Mahony S. M. Clarke G. Borre Y. E. Dinan T. G. Cryan J. F. (2015). Serotonin, tryptophan metabolism and the brain-gut-microbiome axis. Behav. Brain Res. 277, 32–48. 10.1016/j.bbr.2014.07.027 25078296

[B63] Otte C. Gold S. M. Penninx B. W. Pariante C. M. Etkin A. Fava M.. (2016). Major depressive disorder. Nat. Rev. Dis. Primers 2, 16065. 10.1038/nrdp.2016.65 27629598

[B64] Price M. N. Dehal P. S. Arkin A. P. (2010). FastTree 2 – approximately maximum-likelihood trees for large alignments. PLoS ONE 5, e9490. 10.1371/journal.pone.0009490 20224823 PMC2835736

[B65] Ranjan R. Abhinay A. Mishra M. (2018). Can oral microbial infections be a risk factor for neurodegeneration? A review of the literature. Neurol. India 66, 344–351. 10.4103/0028-3886.227315 29547153

[B66] Reinecke A. Filippini N. Berna C. Western D. G. Hanson B. Cooper M. J.. (2015). Effective emotion regulation strategies improve fMRI and ECG markers of psychopathology in panic disorder: implications for psychological treatment action. Transl. Psychiat. 5, e673. 10.1038/tp.2015.160 26529426 PMC5068756

[B67] Rodda J. Walker Z. Carter J. (2011). Depression in older adults. BMJ. 343, d5219. 10.1136/bmj.d5219 21957206

[B68] Rodríguez-Lara A. Plaza-Díaz J. López-Uriarte P. Vázquez-Aguilar A. Reyes-Castillo Z. Álvarez-Mercado A. I. (2022). Fiber consumption mediates differences in several gut microbes in a subpopulation of young mexican adults. Nutrients 14, 1214. 10.3390/nu14061214 35334871 PMC8954685

[B69] Saarimaki H. Gotsopoulos A. Jaaskelainen I. P. Lampinen J. Vuilleumier P. Hari R.. (2016). Discrete neural signatures of basic emotions. Cereb Cortex 26, 2563–2573. 10.1093/cercor/bhv086 25924952

[B70] Scheperjans F. Aho V. Pereira P. A. Koskinen K. Paulin L. Pekkonen E.. (2015). Gut microbiota are related to Parkinson's disease and clinical phenotype. Mov. Disord. 30, 350–358. 10.1002/mds.26069 25476529

[B71] Schmaal L. Hibar D. P. Samann P. G. Hall G. B. Baune B. T. Jahanshad N.. (2017). Cortical abnormalities in adults and adolescents with major depression based on brain scans from 20 cohorts worldwide in the ENIGMA Major Depressive Disorder Working Group. Mol. Psychiatry 22, 900–909. 10.1038/mp.2016.60 27137745 PMC5444023

[B72] Segata N. Izard J. Waldron L. Gevers D. Miropolsky L. Garrett W. S.. (2011). Metagenomic biomarker discovery and explanation. Genome Biol. 12, R60. 10.1186/gb-2011-12-6-r60 21702898 PMC3218848

[B73] Shannon C. E. (1948). A mathematical theory of communication. Bell Syst. Techn. J. 27, 379–423. 10.1002/j.1538-7305.1948.tb01338.x30854411

[B74] Sheehan D. V. Lecrubier Y. Sheehan K. H. Amorim P. Janavs J. Weiller E.. (1998). The Mini-International Neuropsychiatric Interview (M.I.N.I.): the development and validation of a structured diagnostic psychiatric interview for DSM-IV and ICD-10. J Clin Psychiatry 59, 22–33. 9881538

[B75] Sierra M. A. Li Q. Pushalkar S. Paul B. Sandoval T. A. Kamer A. R.. (2020). The influences of bioinformatics tools and reference databases in analyzing the human oral microbial community. Genes 11, 878. 10.3390/genes11080878 32756341 PMC7465726

[B76] Sinclair L. Osman O. A. Bertilsson S. Eiler A. (2015). Microbial community composition and diversity via 16S rRNA gene amplicons: evaluating the illumina platform. PLoS ONE 10, e0116955. 10.1371/journal.pone.0116955 25647581 PMC4315398

[B77] Tan A. H. Lim S. Y. Lang A. E. (2022). The microbiome-gut-brain axis in Parkinson disease - from basic research to the clinic. Nat Rev Neurol. 10.1038/s41582-022-00681-2 [Epub ahead of print]. 35750883

[B78] Tsai C. F. Lee W. J. Wang S. J. Shia B. C. Nasreddine Z. Fuh J. L. (2012). Psychometrics of the Montreal Cognitive Assessment (MoCA) and its subscales: validation of the Taiwanese version of the MoCA and an item response theory analysis. Int. Psychogeriatr. 24, 651–658. 10.1017/S1041610211002298 22152127

[B79] Verhoog S. Taneri P. E. Roa Díaz Z. M. Marques-Vidal P. Troup J. P. Bally L.. (2019). Dietary factors and modulation of bacteria strains of akkermansia muciniphila and faecalibacterium prausnitzii: a systematic review. Nutrients 11, 1565. 10.3390/nu11071565 31336737 PMC6683038

[B80] Walkden H. Delbaz A. Nazareth L. Batzloff M. Shelper T. Beacham I. R.. (2020). Burkholderia pseudomallei invades the olfactory nerve and bulb after epithelial injury in mice and causes the formation of multinucleated giant glial cells in vitro. PLoS Negl. Trop. Dis. 14, e0008017. 10.1371/journal.pntd.0008017 31978058 PMC7002012

[B81] Wickham H. (2009). ggplot2: Elegant Graphics for Data Analysis. New York NY: Springer-Verlag. 10.1007/978-0-387-98141-3

[B82] Wilson R. S. Nag S. Boyle P. A. Hizel L. P. Yu L. Buchman A. S.. (2013). Brainstem aminergic nuclei and late-life depressive symptoms. JAMA Psychiatry 70, 1320–1328. 10.1001/jamapsychiatry.2013.2224 24132763 PMC3856195

[B83] Yoon S. H. Ha S. M. Kwon S. Lim J. Kim Y. Seo H.. (2017). Introducing EzBioCloud: a taxonomically united database of 16S rRNA gene sequences and whole-genome assemblies. Int. J. Syst. Evol. Microbiol. 67, 1613–1617. 10.1099/ijsem.0.001755 28005526 PMC5563544

[B84] Zheng P. Zeng B. Zhou C. Liu M. Fang Z. Xu X.. (2016). Gut microbiome remodeling induces depressive-like behaviors through a pathway mediated by the host's metabolism. Mol. Psychiat. 21, 786–796. 10.1038/mp.2016.44 27067014

